# Development and validation of a new algorithm model for differential diagnosis between Crohn's disease and intestinal tuberculosis: a combination of laboratory, imaging and endoscopic characteristics

**DOI:** 10.1186/s12876-021-01838-x

**Published:** 2021-07-13

**Authors:** Yi Lu, Yonghe Chen, Xiang Peng, Jiayin Yao, Weijie Zhong, Chujun Li, Min Zhi

**Affiliations:** 1 Department of Gastrointestinal Endoscopy, the Sixth Affiliated Hospital, Sun Yat-Sen University, Guangzhou, 510655 People’s Republic of China; 2 Guangdong Provincial Key Laboratory of Colorectal and Pelvic Floor Diseases, The Sixth Affiliated Hospital, Sun Yat-Sen University, 26 Yuancun Erheng Road, Guangzhou, 510655 People’s Republic of China; 3 Department of Gastrointestinal Surgery, the Sixth Affiliated Hospital, Sun Yat-Sen University, Guangzhou, 510655 People’s Republic of China; 4 Department of Gastroenterology, the Sixth Affiliated Hospital, Sun Yat-Sen University, Guangzhou, 510655 People’s Republic of China

**Keywords:** Crohn's disease, Intestinal tuberculosis, Diagnosis, Algorithm, CART

## Abstract

**Background:**

Sometimes in clinical practice, it is a great challenge to distinguish Crohn's disease (CD) and intestinal tuberculosis (ITB), we conducted this study to identify simple and useful algorithm for distinguishing them.

**Methods:**

We retrospectively reviewed the medical history of the patients who were diagnosed as ITB or CD. We firstly identified ITB patients, and then the patients diagnosed with CD were matched by age, sex, and admission time in a 1:1 ratio. Patients who admitted between May 1, 2013 and April 30, 2019 were regarded as training cohort, and patients admitted between May 1, 2019 and May 1, 2020 were regarded as validation cohort. We used multivariate analysis to identify the potential variables, and then we used R package rpart to build the classification and regression tree (CART), and validated the newly developed model.

**Results:**

In total, the training cohort included 84 ITB and 84 CD patients, the validation cohort included 22 ITB and 22 CD patients. Multivariate analysis showed that, positive interferon-gamma release assays (IGRAs), ≥ 4 segments involved, longitudinal ulcer, circular ulcer, and aphthous ulcer were confirmed as independent discriminating factors. Using these parameters to build the CART model made an overall accuracy rate was 88.64%, with sensitivity, specificity, NPV, and PPV being 90.91%, 86.36%, 90.48% and 86.96%, respectively.

**Conclusion:**

We developed a simple and novel algorithm model covering laboratory, imaging, and endoscopy parameters with CART to differentiate ITB and CD with good accuracy. Positive IGRAs and circular ulcer were suggestive of ITB, while ≥ 4 segments involved, longitudinal ulcer, and aphthous ulcer were suggestive of CD.

**Supplementary Information:**

The online version contains supplementary material available at 10.1186/s12876-021-01838-x.

## Introduction

Crohn's disease (CD) and intestinal tuberculosis (ITB) are two intestinal diseases with different etiology, pathogenesis, and treatment. Sometimes in clinical practice, it is a great challenge to distinguish the two diseases. It was reported that the misdiagnosis rate between the two diseases could reach 50%-70% [1]. The diagnosis of ITB can be made when acid fast bacilli (AFB) and granulomas with caseous necrosis are found in histopathology, but the positive rates are low [2, 3]. If ITB could not be excluded, 8–12 weeks of empirical antituberculosis treatment (ATT) is recommended, avoiding the severe adverse events if immunosuppressive drugs are prescribed to ITB patients by mistake [4, 5]. Nevertheless, the ATT can delay CD treatment, and ATT has many side effects [5, 6]. Hence, models that can be used at the bedside for easy and accurate differential diagnosis would be extremely useful in clinical practice. As a result, many researchers have tried to identify parameters and models that could improve the accuracy rate [7–9]. For laboratory parameters, interferon-gamma release assays (IGRAs, including T-SPOT and QuantiFERON-TB Gold) were important in differentiating ITB and CD, and a positive result was more likely to be ITB [10]. For imaging characteristics, target sign, comb sign, adipose creeping sign and involvement of fewer than 4 segments were reported to be vital parameter, and the former three were more like to be the characteristics of CD, while the last was more likely to be ITB [8, 9, 11–13]. For endoscopic parameters, longitudinal ulcers, transverse ulcers, aphthous ulcers, cobblestone, and stricture were proved to be useful, and transverse ulcers were specific to ITB, while the others were more likely to be CD [14]. There parameters are of various kinds, and the scoring systems also vary from each other, and some models are complex to use. Therefore, we conducted this study to investigate the laboratory, imaging and endoscopic characteristics of CD and ITB, and to identify simple and useful algorithm for distinguishing the two diseases.

## Methods

### Patients

We retrospectively reviewed the medical history of the patients who were diagnosed as ITB or CD between May 1, 2013 and May 1, 2020. The inclusion criteria were: (1) had results of IGRAs; (2) had undergone colonoscopy or trans-anal enteroscopy in our hospital so as to obtain the endoscopic characteristics; (3) had the results of computed tomography enterography (CTE) or magnetic resonance enterography (MRE) so as to obtain imaging characteristics; (4) the diagnosis was not confirmed when the patients visited our hospital; (5) the patients had not received any treatment or had received ATT or glucocorticoids or immunosuppressive agents for no more than 1 week; (6) the diagnosis of either ITB or CD could be confirmed. The exclusion criteria were: (1) the result of IGRAs or endoscopic characters or CTE/MRE was not complete; (2) had received treatments for more than 1 week; (3) final diagnosis could not be confirmed; (4) ITB and CD both existed; (5) had colectomy before this hospitalization.

### Diagnostic criteria

The confirmed diagnosis for ITB required one of the following: (a) caseating granulomas detected in endoscopy biopsy, surgical specimen, or mesenteric lymph nodes; (b) demonstration of AFB on smears or histological sections or positive culture for AFB; (c) strong suspicion of ITB with a good response to ATT without recurrence; a good response to ATT was determined by relief of symptoms and disappearance of ulcerations on endoscopic examination for at least 6 months of follow-up [7, 8, 15].

The confirmed diagnosis for CD was made based on the management consensus of inflammatory bowel disease for the Asia–Pacific region [16], which included clinical, endoscopic, histological, and radiological characteristics and/or biochemical evaluation.

### Study design

Firstly, we identified the patients diagnosed with ITB who met all the inclusion criteria (group ITB). Then the patients diagnosed with CD were matched by age, sex, and admission time in a 1:1 ratio (group CD). We reviewed the medical history of the patients including age, sex, weight, height, symptoms, present and past history, laboratory results [blood routine, erythrocyte sedimentation rate (ESR), albumin, hsCRP, stool routine, IGRAs and PPD skin test], endoscopy results (location of lesions, shape of ulcers, cobblestone appearance, pseudopolyp, scar, and stricture), imaging results (enlargement of celiac lymph nodes, peritoneal abscess, bowel wall thickening, stricture, comb sign, target sign, adipose creeping sign [13], ≥ 4 segments involved, intestinal fistula, perianal diseases and chest X-ray/CT), pathology results (acid fast bacilli staining, granulomas, caseous necrosis or non-caseous necrosis), diagnosis, treatment and follow-up results. The definitions of each laboratory, endoscopic and imaging finding are presented in the Additional file 1 [11–13, 17, 18]. Patients who admitted between May 1, 2013 and April 30, 2019 were regarded as training cohort, the information of which was used to develop the algorithm; and patients admitted between May 1, 2019 and May 1, 2020 were regarded as validation cohort, the information of which was used to validate the algorithm. The study was approved by the ethics committee in our hospital.

### Statistical analysis

IBM SPSS Statistic Version 24.0.0.0 was used to compare the differences between the two groups. Continuous variables without normal distribution were presented with median (lower and upper interquartile range), and tested by nonparametric Wilcoxon rank-sum test. Categorical variables were presented with number (percentage), and were tested by χ^2^ test. Statistical significance was defined as *P* < 0.05 (two-tailed). After the comparison, we identified some parameters which had statistical differences between the two groups, and selected them as potential variables to build the algorithm.

The study end point was to develop an algorithm to differentiate ITB and CD, and to validate it. One hundred and sixty-eight and forty-four cases were allocated to the training cohort and validation cohort, respectively. Parameters with statistical significance were enrolled into logistic regression for multivariate analysis. Independent discriminating factors selected by the logistic model were then used to build a decision tree based on the R package rpart (https://CRAN.R-project.org/package=rpart). Briefly, this is a nonparametric regression method based on binary recursive partitioning of data in the training set to build a model like a tree structure, which is also called classification and regression tree (CART). It incrementally divides the data into smaller subclasses according to the amount of information gained by each partition into a subclass until no additional information could be gained by a split [19]. It generated a tree structure flow chart to differentiate ITB and CD that is easy to interpret. The overall sensitivity, specificity, negative predictive value (NPV), positive predictive value (PPV) and predictive accuracy of the CART were evaluated using data of the validation cohort. *P* < 0.05 was regarded as statistical significance. R version 4.0.2 (R Foundation for Statistical Computing, Vienna, Austria. https://www.R-project.org) was used to perform the statistical analysis.

## Results

### Baseline and laboratory characteristics of the training cohort

In total, the training cohort included 84 ITB and 84 CD patients, the validation cohort included 22 ITB and 22 CD patients. In the training cohort, 31 patients (36.90%) have received empirical ATT in group ITB; the ratio of patients with diarrhea, symptoms of perianal diseases (perianal pain, perianal discomfort, perianal pus or fluids), anemia, PLT > 300*10^9/L, hsCRP > 3 mg/L, and positive occult blood test was statistically higher in group CD than that in group ITB; the ratio of positive PPD skin test and positive IGRAs was higher in group ITB (The details are shown in Table 1).

**Table 1:** Clinical and laboratory characteristics of the training cohort

	ITB (n = 84)	CD (n = 84)	P value
Age, years	32 (24–46)	32 (26–46)	0.685
Male, n (%)	52 (61.90)	52 (61.90)	1
*Symptoms*			
Abdominal pain	33 (39.29)	32 (38.10)	0.874
Diarrhea	8 (9.52)	33 (39.29)	< 0.0001
Gastrointestinal bleeding	8 (9.52)	11 (13.10)	0.465
Weight loss	33 (39.29)	25 (29.76)	0.194
Fever	5 (5.95)	2 (2.38)	0.443
Perianal diseases	11 (13.10)	33 (39.29)	< 0.0001
History of appendicectomy	6 (7.14)	4 (4.76)	0.514
Anemia	21 (25)	33 (39.29)	0.047
PLT > 300*10^9/L	29 (34.52)	47 (55.95)	0.005
WBC > 10*10^9/L	10 (11.90)	12 (14.29)	0.647
ESR > 20 mm/h^#^	52 (61.90)	55 (66.27)	0.557
Hypoalbuminemia	19 (22.62)	29 (34.52)	0.088
hsCRP > 3 mg/L^#^	42 (50.60)	65 (78.31)	< 0.0001
Positive occult blood test^#^	18 (21.69)	29 (36.25)	0.04
Positive PPD skin test^#^	57 (68.67)	9 (12.5)	< 0.0001
Positive IGRAs	76 (90.48)	12 (14.29)	< 0.0001

ITB, intestinal tuberculosis; CD, Crohn's disease; PLT, platelet; WBC, white blood cell; ESR, erythrocyte sedimentation rate

^#^ Not all patients had the result

### Imaging characteristics of the training cohort

The CTE/MRE results analysis showed that, the ratio of patients with comb sign, target sign, adipose creeping sign and ≥ 4 segments involved was significantly higher in group CD than that in group ITB. Anal magnetic resonance image (MRI) results showed that more patients in group CD have anal fistula or perianal abscess. Chest X-ray/CT results showed that more patients in group ITB had pulmonary TB and calcification (The imaging characteristics of the training cohort are shown in Table 2).

**Table 2:** Imaging characteristics of the training cohort

	ITB (n = 84)	CD (n = 84)	P value
*CTE/MRE, n (%)*			
Enlargement of celiac lymph nodes	30 (35.71)	25 (29.76)	0.411
Peritoneal abscess	2 (2.38)	7 (8.33)	0.087
Bowel wall thickening	81 (96.43)	82 (97.62)	0.65
Stricture	19 (22.62)	29 (34.52)	0.088
Comb sign	15 (17.86)	60 (71.43)	< 0.0001
Target sign	29 (34.52)	62 (73.81)	< 0.0001
Adipose creeping sign	7 (8.33)	31 (36.9)	< 0.0001
≥ 4 segments involved	15 (17.86)	68 (80.95)	< 0.0001
Intestinal fistula	2 (2.38)	6 (7.14)	0.147
*Anal MRI*^*#*^			
Anal fistula or perianal abscess	13 (39.39)	52 (86.67)	< 0.0001
*Chest X-ray/CT*			
Pulmonary TB	34 (40.48)	0 (0)	< 0.0001
Fibrosis	22 (26.19)	14 (16.67)	0.133
Calcification	12 (14.29)	4 (4.76)	0.035
Infection^*^	7 (8.33)	3 (3.57)	0.192

ITB, intestinal tuberculosis; CD, Crohn's disease; CTE, computed tomography enterography; MRE, magnetic resonance enterography; MRI, magnetic resonance image; CT, computed tomography; TB, tuberculosis

^*^Infection but not sufficient to support TB infection; ^#^ not all patients had the result

### Endoscopy and pathology characteristics of the training cohort

**Fig. 1:**
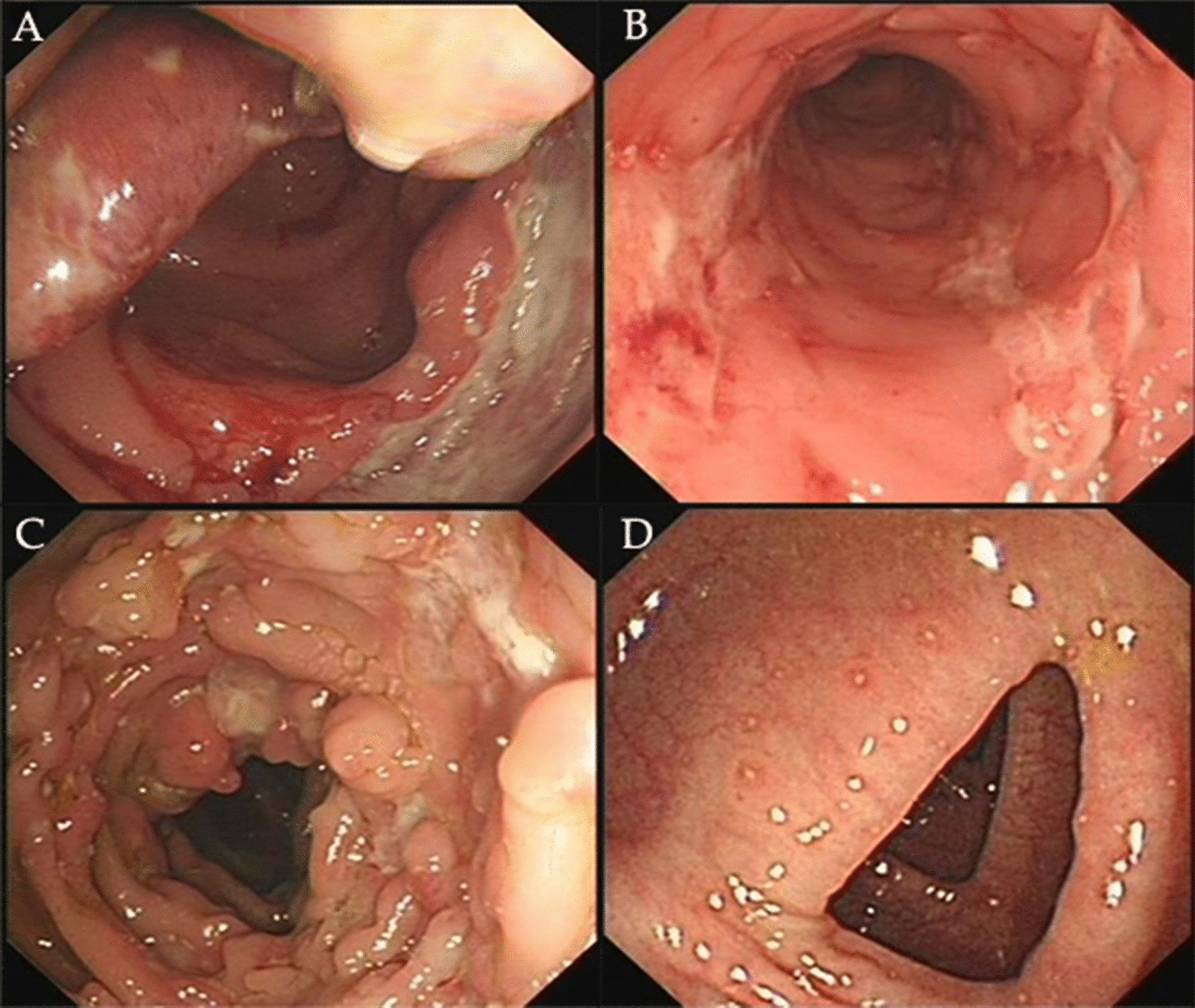
Endoscopy images of **a** circular ulcer; **b** longitudinal ulcer; **c** cobblestone appearance; **d** aphthous ulcer

The endoscopy results showed that, the ratio of patients with longitudinal ulcer, aphthous ulcer (the endoscopy images are shown in Fig. 1), rectum involvement, and sigmoid colon involvement was significantly higher in group CD than that in group ITB. More patients in group ITB had circular ulcer, and the pathology result showed that, the ratio of caseous necrosis was also higher in group ITB (The endoscopy and pathology characteristics of the training cohort are shown in Table 3).

**Table 3:** Endoscopy and pathology characteristics of the training cohort

	ITB (n = 84)	CD (n = 84)	P value
*Endoscopy, n (%)*			
Irregular ulcer	56 (66.67)	71 (60.71)	0.422
Longitudinal ulcer	8 (9.52)	30 (35.71)	< 0.0001
Circular ulcer	26 (30.95)	1 (1.19)	< 0.0001
Aphthous ulcer	2 (2.38)	35 (41.67)	< 0.0001
Cobblestone appearance	4 (4.76)	11 (13.10)	0.058
Pseudopolyp	35 (41.67)	32 (38.10)	0.636
Scar	8 (9.52)	12 (14.29)	0.341
Stricture	24 (28.57)	19 (22.62)	0.377
Fistula	1 (1.19)	2 (2.38)	1
Rectum involvement	11 (13.10)	33 (39.29)	< 0.0001
Sigmoid colon involvement	11 (13.10)	31 (36.90)	< 0.0001
*Pathology*			
Granulomas	62 (73.81)	52 (61.90)	0.099
Caseous necrosis	8 (9.52)	0 (0)	0.007
Non-caseous necrosis	56 (66.67)	64 (76.19)	0.172
Positive AFB staining^#^	4 (16)	0 (0)	1

ITB, intestinal tuberculosis; CD, Crohn's disease; AFB, acid fast bacilli

^#^ Not all patients had the result

### Multivariate analysis and diagnostic values of the factors

When these parameters were entered into a multivariate analysis, positive IGRAs, ≥ 4 segments involved, longitudinal ulcer, circular ulcer, and aphthous ulcer were confirmed as independent discriminating factors (Table 4 shows the multivariate analysis results). Further analysis of the diagnostic values of these factors is shown in Table 5. The most sensitive and specific parameters for differentiating ITB and CD when used in isolation were circular ulcer (sensitivity 100%) and longitudinal ulcer (specificity 100%). However, circular ulcer was not specific for ITB, because 50% of CD patients had a circular ulcer. Similarly, longitudinal ulcer was specific to CD, but the true positive rate was only 40.91% in CD patients.

**Table 4:** Multivariate analysis to identify the independent discriminating factors

Factors	Estimate	Standard error	Z value	P value
Intercept	− 0.05	0.64	− 0.083	0.934
Positive IGRAs	− 3.62	0.77	− 4.672	< 0.0001
≥ 4 segments involved	2.87	0.82	3.499	0.0005
Longitudinal ulcer	2.06	0.93	2.225	0.026
Circular ulcer	− 2.52	1.25	− 2.022	0.043
Aphthous ulcer	2.52	1.21	2.089	0.037
Comb sign/ target sign/ Adipose creeping sign	0.53	0.72	0.742	0.458
Rectum involvement	− 0.31	0.94	− 0.325	0.745

**Table 5:** Accuracy of using each single discriminating factor in differentiating ITB and CD

Factors	Sensitivity, %	Specificity, %	PPV, %	NPV, %
Positive IGRAs	86.36	82.82	82.61	85.71
≥ 4 segments involved	81.82	77.27	78.26	80.95
Longitudinal ulcer	40.91	100	100	62.86
Circular ulcer	100	50	66.67	100
Aphthous ulcer	54.55	90.91	85.71	66.67

ITB, intestinal tuberculosis; CD, Crohn's disease; PPV, positive predictive value; NPV, negative predictive value

### The CART analysis of training and validation cohorts

**Fig. 2:**
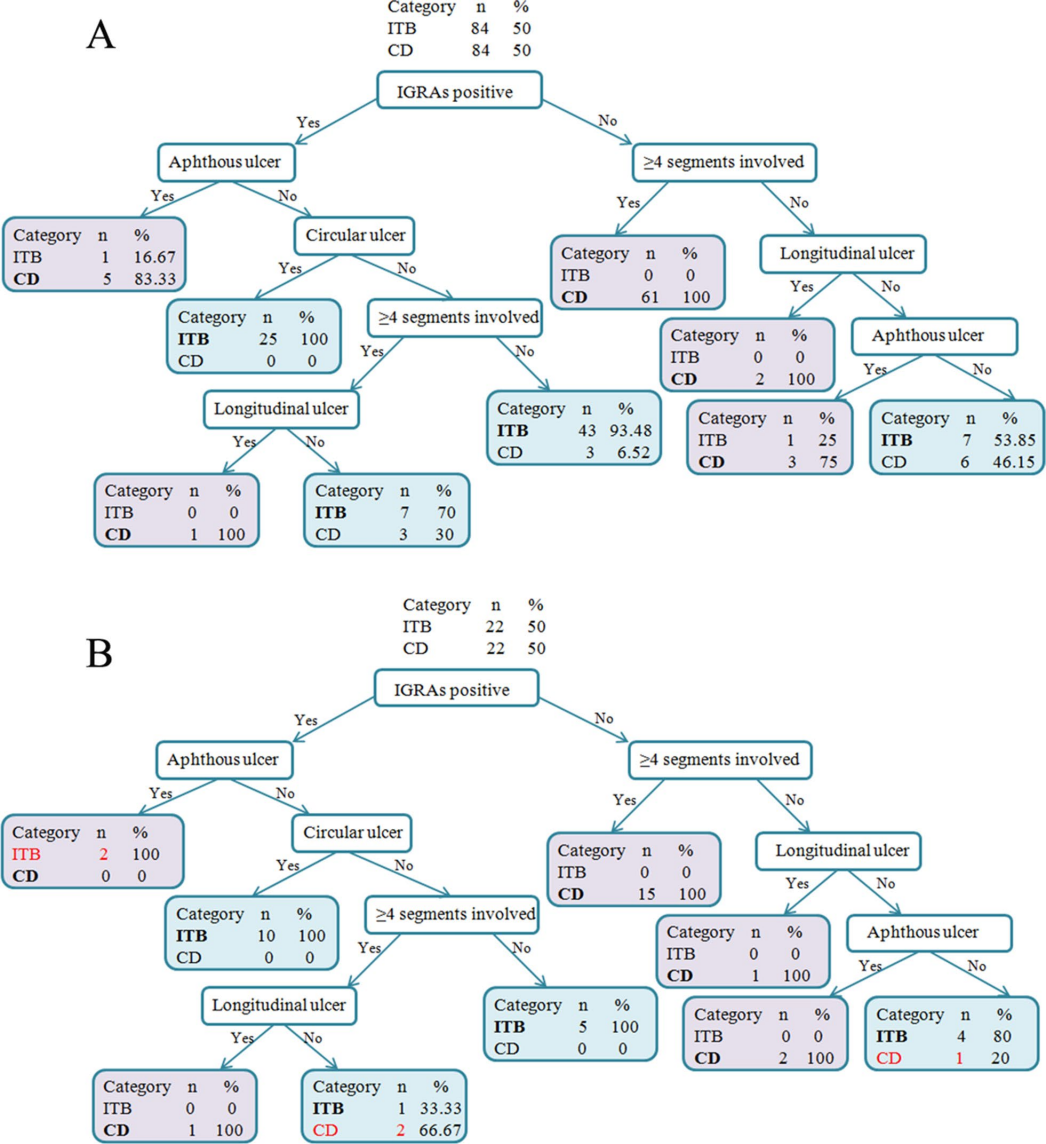
**a** The classification and regression tree (CART) analysis in the training cohort; **b** the CART analysis in the validation cohort (the purple background stands for diagnosis of CD, green background stands for diagnosis of ITB, and the words in red color stands for the misdiagnosed patients using this model). ITB, intestinal tuberculosis; CD, Crohn’s disease; IGRAs, interferon-gamma release assays

The first factor that split the training population was IGRAs. 86.36% of the patients with positive IGRAs were diagnosed as ITB, while 90% of the patients with negative IGRAs were diagnosed as CD. The CART analysis result for the training cohort is shown in Fig. 2a. From the figure, we could find that, patients with positive IGRAs and circular ulcer but without aphthous ulcer have high reliability in the diagnosis of ITB; patients with negative IGRAs and with ≥ 4 segments involved/ longitudinal ulcer have high reliability in the diagnosis of CD.

When we tested this algorithm using the data in the validation cohort, the overall accuracy rate was 88.64% (39 in 44 patients). The CART analysis in the validation cohort is shown in Fig. 2b. The sensitivity, specificity, NPV, and PPV of differential diagnosis of ITB from CD was 90.91%, 86.36%, 90.48% and 86.96%, respectively (Table 6).

**Table 6:** Differential diagnosis based on the classification and regression tree in validation cohort

Predicted category (n)	Actual category		Total
	ITB (n = 22)	CD (n = 22)	
ITB	20	3	23
CD	2	19	21
Total	22	22	44

ITB, intestinal tuberculosis; CD, Crohn's disease

## Discussion

Differentiating ITB from CD is sometimes very difficult in some patients, especially in the areas where both ITB and CD are prevalent, as there are many similarities between ITB and CD in clinical manifestations, laboratory results, imaging, endoscopic and even histological characteristics. On the other hand, the misdiagnosis or delayed diagnosis can result in tubercle bacillus diffusion, drug adverse events and medical expenditure increase [20]. Hence, there is an urgent need for developing accurate and easy diagnostic tools to differentiate ITB and CD. Many previous studies have been conducted to construct models that could improve the accuracy [7–9, 11, 21], but they used different diagnostic models and scoring systems, and some of them were not easy to use, as they may need a calculator or a computer. In this study, we finally selected five parameters (positive IGRAs, ≥ 4 segments involved, longitudinal ulcer, circular ulcer, and aphthous ulcer) covering laboratory, imaging and endoscopic characteristics, and constructed the CART algorithm, which could be used at the bedside for easy and accurate differential. To our knowledge, this is the first study to use CART to differentiate ITB and CD.

In this study, we firstly compared the clinical, imaging, endoscopic and pathologic characteristics between ITB and CD in the training cohort. For clinical characteristics in the training cohort, patients with CD were more likely to have diarrhea, and symptoms of perianal diseases, this was in accordance with previous studies [7, 21]. For laboratory characteristics, patients with CD were more likely to have anemia, PLT > 300*10^9/L, hsCRP > 3 mg/L, and positive occult blood test than ITB, the results were close to some of the previous studies, but some were not in consistency [7, 9, 21]. Patients with ITB had much higher rate of positive IGRAs, which was a very important parameter in the CART, as it split the patients in the first step. A meta-analysis showed that, the pooled sensitivity and specificity of IGRAs for the differential diagnosis of ITB from CD were 82.8% and 86.7%, respectively, indicating that it was a good independent discriminating factor [10]. In this study and in clinical, some patients had a positive TB test but the final diagnosis was CD, the algorithm helped us to differentiate this as follows: if they also had aphthous ulcer, then the probability of CD was about 83.33%; if they did not have aphthous ulcer no circular ulcer, but have ≥ 4 segments involved and longitudinal ulcer, then probability of CD was very high, otherwise, the diagnosis would incline to ITB.

For imaging characteristics, especially CTE/MRE, patients with CD were more likely to show comb sign, target sign, adipose creeping sign and ≥ 4 segments involved. Kedia et al. showed that comb sign was more common in CD [11], and no studies had compared target sign or adipose creeping sign in ITB and CD, but some studies have used them to build the differential models [8, 12]. In our study, these three factors were probably easy to get influenced by other factors, and they failed to be selected as potential parameters in CART. Except for these signs in CTE, Kedia et al. also evaluated other characteristics in CTE, and they found that, necrotic lymph nodes were specific for ITB, while the combination of long segment lesion and visceral to subcutaneous fat ratio (VF/SC) ratio > 0.63 was specific for CD, using these characters could make an accuracy rate of 43%[11].

For endoscopic characteristics, rectum or sigmoid colon involvement and longitudinal ulcers, aphthous ulcers were more common in CD, whereas transverse ulcers were more common in ITB, our results is the same as the previous published ones [9, 14, 21]. Lee et al. selected eight endoscopic characteristics along to build the models: transverse ulcers, less than 4 segment lesions, patulous ileocecal valve, and pseudopolyp (favored ITB); anorectal lesions, aphthous ulcers, longitudinal ulcers, and cobblestone appearance (favored CD), and they hypothesized that if there were more parameters favored of ITB than CD, then a diagnosis of ITB could be made, and vice versa. This endoscopic model could make an accuracy rate of 87.5% and the PPV for CD and ITB was 95% and, respectively, indicating that a systematic analysis of endoscopic findings is very useful in differentiating ITB and CD [22]. In our study, we found that the shapes of the ulcers were very important parameters in differentiating ITB and CD, and transverse ulcers, longitudinal ulcers, and aphthous ulcers were all selected in our CART model. Regarding the importance of the shapes of the ulcers, we suppose that it may be possible to use the artificial intelligence (AI) technique, which is now very popular, to help in differentiating ITB and CD. As it can be taught to identify the characteristics of the ulcers (such as the shapes), and if we further enter some other parameters, after calculation and combination, the AI might give us a satisfied answer.

For pathologic characteristics, granulomas and non-caseous necrosis could be seen in both groups, while caseous necrosis and positive AFB staining was exclusive to ITB, but the sensitivity was low. Qian et al. compared granulomatous lymphangitis and granulomas in CD and ITB, and they concluded that, neither granulomatous lymphangitis nor granulomas was specific for CD, but their morphology and distribution could help in the differential diagnosis [23]. In our study, the pathologic characteristics were not selected as parameters to build the CART model, but we suppose that, if we study the morphology and distribution of granulomas next time, we may find some meaningful parameters.

Using a single parameter to distinguishing ITB and CD has very limited use in clinical practice, as its sensitivity or specificity is usually low. Hence, an integration of various categories of valuable parameters to establish a model could possibly help to deal with this question. Mao et al. selected 2 CTE findings and 8 endoscopic findings to build the model, and they found that the accuracy rate increased from 71.6 to 88.3%, compared with using endoscopic findings alone [24]. Bae et al. also developed a scoring system using 8 endoscopic findings, chest ray, small bowel follow through, and 2 laboratory tests [anti-Saccharomyces cerevisiae antibodies (ASCA) and IGRAs], which showed very good results, with an accuracy rate of 96.3% [9]. But there is limitation in their model, as in our hospital and many other hospitals in China, ASCA is not routine tested, we may fail in using this model. Some studies used likelihood ratio models to differentiate these two diseases [7, 25], and their accuracy rate could reach 95.7%. And there were many other models used as Limsrivilai et al. showed in their systematic review [5], but these models usually contained a very complex formula, and need a calculator, so they are difficult to be applied at the bedside. CART can dissect complicated clinical situations and identifies homogeneous patient groups, so it is able to generate clinical decision trees. In our CART model, the parameters are easy to obtain, and does not need any calculation, we think it will be more convenient to use. As in the previous models, they usually used logistic regression or nomogram which calculated a probability of disease, were inconvenient for clinicians, as they generally think in terms of categories, such as “yes” or “no”, rather than think about probability. So the CART model is ideally suited to generation of decision trees for the clinicians.

There are some limitations in this study. Firstly, this is a single-centered, retrospective study, and the sample size is limited, and due to its retrospective, some other factors could not be assessed, on the other hand, though we validated this algorithm, it was still internal validation, so prospective, larger sample-sized, and multi-centered study is still needed to make it more convincible and can be applied to other centers. Secondly, the patients with CD were matched by age, sex, and admission time in a 1:1 ratio, while the former two elements might be important parameters in other studies [8, 21], while we did not include these parameters, as we planned to build the CART, and the variables used to build the model were categorical variables, as age was not categorical variable, and we cannot find a cut-off point younger than which was CD, so age was not used to enter the model. As for gender, we thought it was impossible to differentiate the two diseases based on gender in the CART model, so we matched the patients of the same age and gender, and focused more on other variables we were interested in. Thirdly, the diagnostic algorithm started with IGRAs result, while in clinical practice, sometimes we might have indeterminate result, than it was not suitable to use this model. Actually, this usually occurs in immunosuppressed patients, and the incidence was reported to be 0% to 9.7% [26–28], though not high, it was really a thorny problem in clinical. In the future work, we must investigate how to deal with patients with indeterminate result. Fourthly, CART itself also has disadvantages, for example, in some branches, the accuracy rate was not that satisfied, and that as CART is not based upon a probabilistic model, so the CART tree cannot provide the probability level or confidence interval of the predictions [29, 30].

In conclusion, we developed a simple and novel algorithm model covering laboratory, imaging, and endoscopy parameters with CART to differentiate ITB and CD with good accuracy, but external validation is warranted. Except for our model, many models have been proposed in the previous published studies, and we can select the model that is most appropriate and easy to apply.

## Supplementary Information

**Additional file 1.** Definitions of the Laboratory, Imaging and Endoscopic Findings.

## Data Availability

The datasets used are available from the corresponding author on reasonable request.

## Abbreviations

CD: Crohn's disease
ITB: Intestinal tuberculosis
CART: Classification and regression tree
AFB: Acid fast bacilli
ATT: Antituberculosis treatment
CTE: Computed tomography enterography
MRE: Magnetic resonance enterography
ESR: Erythrocyte sedimentation rate
NPV: Negative predictive value
PPV: Positive predictive value
AI: Artificial intelligence
ASCA: Anti-Saccharomyces cerevisiae antibodies
