# Supplementary material for: Development and validation of a new algorithm model for differential diagnosis between Crohn's disease and intestinal tuberculosis: a combination of laboratory, imaging and endoscopic characteristics
Source: BMC Gastroenterol. 2021 Jul 13;21:291. doi: 10.1186/s12876-021-01838-x (PMC8276438; doi:10.1186/s12876-021-01838-x)
Supplement: Supplementary file 1 — Additional file 1. Definitions of the Laboratory, Imaging and Endoscopic Findings. [file 12876_2021_1838_MOESM1_ESM.docx]

**Definitions of the Laboratory, Imaging and Endoscopic Findings**

**Laboratory:**

Positive PPD skin test: 1 mL tuberculin purified protein derivative and 1 mL physiological saline solution were used. Using a 1 mL syringe, 0.1 mL concentrate was removed and diluted with saline to 0.25 mL, 0.1mL of which was then intradermally into the medial forearm of the patient. The result is positive (+) if thescleroma diameter is > 5 mm but ≤ 10 mm; positive (++) if the scleroma diameter is > 10 mm but ≤ 20 mm; strongly positive (+++) if the scleroma diameter is ＞ 20 mm or has local blisters, necrosis or lymphangitis, and in this study, we regarded the above situations as positive; if the scleroma diameter is less than 5 mm then is negative.

Interferon-gamma release assays (IGRAs): We have used two kinds of IGRAs, before November, 2019 the blood samples were sent to Huayin Medical Laboratory Center, and they used QuantiFERON-TB Gold method (reagents produced by QIAGEN company in Germany).after that, the samples were tested in our hospital, and we used T-SPOT method (reagents produced by Autobio company in China). The cut-off values are shown in the following tables:

QuantiFERON-TB Gold:

| N (IU/mL) | T-N | P-N | Result |
| --- | --- | --- | --- |
| ≤8 | <0.35 | ≥0.5 | Negative |
|  | ≥0.35 and <25%N | ≥0.5 |  |
|  | ≥0.35 and ≥25%N | Any | Positive |
|  | <0.35 | <0.5 | Indeterminate |
|  | ≥0.35 and <25%N | <0.5 |  |
| >8 | Any | Any |  |

T-SPOT:

| N (IU/mL) | T-N | P-N | Result |
| --- | --- | --- | --- |
| ≤10 | <0.438 | ≥0.625 | Negative |
|  | ≥0.438 and <25%N | ≥0.625 |  |
|  | ≥0.438 and ≥25%N | Any | Positive |
|  | <0.438 | <0.625 | Indeterminate |
|  | ≥0.438 and <25%N | <0.625 |  |
| >10 | Any | Any |  |

N: the value of negative control, T: the value of tested samples; P: the value of positive control.

**Imaging:**

Enlargement of celiac lymph nodes: lymph nodes with short axis≥10mm.

Bowel wall thickening : bowel wall > 3 mm of an adequately distended loop.

Stricture: lack of bowel loop distention with prestenotic dilatation.

Comb sign: hypervascularity of the mesentery with vascular dilatation, and prominence of the vasa recta.

Target sign: mural stratification, bilaminar appearance of the bowel wall with mucosal hyperenhancement and intramural hypoenhancement or a trilaminar appearance with mucosal and serosal hyperenhancement and intramural hypoenhancement.

Adipose creeping sign: Focal or regionally abnormal prominent mesenteric fat adjacent to affected bowel loops.

Intestinal fistula: abnormal communication between two epithelial surfaces or from the bowel wall.

**Endoscopic:**

Irregular ulcer: the shape of the ulcer is irregular, and cannot be ascribed to longitudinal ulcer, circular ulcer, or aphthous ulcer.

Longitudinal ulcer: ulcer distributing along with the long axis of the bowel, and the longitudinal axis is longer than the transverse axis.

Circular ulcer: round or transverse, deep and well‑demarcated ulcer, and the transverse axis is longer than the longitudinal axis.

Aphthous ulcer: small (<1 cm), round, punched out, raised or a flat lesion with a white center.

Cobblestone appearance: mucosal with raised nodules, resembling the paving of the “Roman” road.

Pseudopolyp: polypoid lesions isolated or multiple and scattered, which are usually small and sometimes can be big in size.

Stricture: presence of luminal narrowing that prevented the passage of the colonoscope in the bowel.

Fistula: abnormal fistula orifice observed in the epithelial surfaces of the bowel wall during endoscopic procedure.
